# High‐resolution synchrotron imaging shows that root hairs influence rhizosphere soil structure formation

**DOI:** 10.1111/nph.14705

**Published:** 2017-07-31

**Authors:** Nicolai Koebernick, Keith R. Daly, Samuel D. Keyes, Timothy S. George, Lawrie K. Brown, Annette Raffan, Laura J. Cooper, Muhammad Naveed, Anthony G. Bengough, Ian Sinclair, Paul D. Hallett, Tiina Roose

**Affiliations:** ^1^ Bioengineering Sciences Research Group Engineering Sciences Academic Unit Faculty of Engineering and the Environment University of Southampton Southampton SO17 1BJ UK; ^2^ Ecological Sciences Group The James Hutton Institute Invergowrie Dundee DD2 5DA UK; ^3^ Institute of Biological and Environmental Science University of Aberdeen Aberdeen AB24 3UU UK; ^4^ School of Science and Engineering University of Dundee Dundee DD1 4HN UK

**Keywords:** *Hordeum vulgare*, image‐based modelling, noninvasive imaging, rhizosphere, root hairs, soil structure, synchrotron

## Abstract

- In this paper, we provide direct evidence of the importance of root hairs on pore structure development at the root–soil interface during the early stage of crop establishment.
- This was achieved by use of high‐resolution (*c*. 5 μm) synchrotron radiation computed tomography (SRCT) to visualise both the structure of root hairs and the soil pore structure in plant–soil microcosms. Two contrasting genotypes of barley (*Hordeum vulgare*), with and without root hairs, were grown for 8 d in microcosms packed with sandy loam soil at 1.2 g cm^−3^ dry bulk density. Root hairs were visualised within air‐filled pore spaces, but not in the fine‐textured soil regions.
- We found that the genotype with root hairs significantly altered the porosity and connectivity of the detectable pore space (> 5 μm) in the rhizosphere, as compared with the no‐hair mutants. Both genotypes showed decreasing pore space between 0.8 and 0.1 mm from the root surface. Interestingly the root‐hair‐bearing genotype had a significantly greater soil pore volume‐fraction at the root–soil interface.
- Effects of pore structure on diffusion and permeability were estimated to be functionally insignificant under saturated conditions when simulated using image‐based modelling.

## Introduction

Plant roots use a range of mechanisms to alter the physical properties of the soil adjacent to roots known as the rhizosphere (Hinsinger *et al*., 2009). Various soil physical stresses and interactions occur during root growth that can be affected by a range of root traits (Bengough *et al*., 2011). Soil compaction around roots has been extensively studied (Dexter, 1987; Bruand *et al*., 1996; Young, 1998; Vollsnes *et al*., 2010; Aravena *et al*., 2011, 2014). Based on these studies the rhizosphere is expected to have both less porosity and smaller pore sizes than bulk soil. However, as roots mature, soil structure is significantly altered by the interplay between root exudates, microbial activity and variations in soil water potential (Hinsinger *et al*., 2009). Consequently, soil in the rhizosphere may have similar or greater porosity and larger pore sizes than bulk soil (Whalley *et al*., 2005; Feeney *et al*., 2006; Hallett *et al*., 2009).

Rhizosphere soil can form a rhizosheath, a layer of strongly bound and more aggregated soil that adheres firmly to the root surface. The size and adherence of the rhizosheath varies significantly between species (Brown *et al*., 2017), and between genotypes of the same species (George *et al*., 2014; Delhaize *et al*., 2015). The formation of a rhizosheath is thought to be driven by root exudates and soil water regime (Watt *et al*., 1994), and by the presence of root hairs (Haling *et al*., 2010, 2014). Some root and microbially derived exudates affect soil structure by binding soil particles and increasing the stability of the rhizosphere (Czarnes *et al*., 2000; Hallett *et al*., 2009). Aggregation of soil particles results from the interplay between these exudates and wetting–drying cycles imposed by plant transpiration (Albalasmeh & Ghezzehei, 2014). Caravaca *et al*. (2005) found that plant species and rhizosphere microbial community affected aggregate stability. Moreno‐Espíndola *et al*. (2007) showed that root hairs increased soil adhesion to roots in sandy soils. These results emphasise the importance of plant genotype on rhizosphere formation. While there has been a wealth of research on how plant genotype affects the rhizosphere microbial community (Ehrenfeld *et al*., 2005; Berg & Smalla, 2009), a thorough understanding of the physical function of the rhizosphere has lagged behind. There is, for instance, an ongoing debate as to whether rhizosphere soil can hold more water than bulk soil (Carminati *et al*., 2010). There is evidence for both lower (Brown *et al*., 1990; Grose *et al*., 1996; Daly *et al*., 2015) and higher water content in the rhizosphere compared to bulk soil (Young, 1995; Carminati *et al*., 2010). This is partly due to the difficulty of disentangling the biophysical and chemical factors that drive rhizosphere function. Additionally, rhizosphere properties are dynamic in time and depend upon root age (Hinsinger *et al*., 2005; Carminati & Vetterlein, 2013). The structure of the pore space around roots has major implications for hydraulic properties, gas permeability and microbial habitats. Therefore, there is clearly potential for plant breeders to select genotypes with improved root traits (White *et al*., 2013).

One set of root traits that offers significant potential for breeding is the density and length of root hairs (Brown *et al*., 2013). Root hairs are thought to improve soil penetration and root soil contact (Haling *et al*., 2013; Bengough *et al*., 2016). It is also commonly estimated that they play a major role in efficient phosphorus uptake, particularly under limited P availability (Bates & Lynch, 2001; Brown *et al*., 2013; Haling *et al*., 2013; Keyes *et al*., 2013). The density and length of root hairs shows considerable variability in response to P availability (Bates & Lynch, 1996; Ma *et al*., 2001), soil water regime and soil compression (Haling *et al*., 2014). Despite their role in exudation (Head, 1964; Czarnota *et al*., 2003) and their potential impact on microbial community structure (Bulgarelli *et al*., 2012, 2013), the impact of root hairs on soil structure has received little attention. There is, however, evidence that root hairs increase soil aggregation (Moreno‐Espíndola *et al*., 2007) and are closely linked to rhizosheath formation (George *et al*., 2014; Haling *et al*., 2014; Delhaize *et al*., 2015).

Root hair interactions with soil structure can now be investigated *in situ* with sufficient resolution due to recent advances in noninvasive synchrotron radiation computed tomography (SRCT). Keyes *et al*. (2013) used SRCT to image living root hairs growing in soil. The three‐dimensional (3D) root and soil images can be used to build numerical models of water and solute movement, enabling soil structural changes to be linked to root uptake functions. The combination of noninvasive imaging and mathematical modelling has been used to understand the effect of root‐induced compaction on water flow in the rhizosphere (Aravena *et al*., 2011, 2014). Daly *et al*. (2015) used image‐based modelling to assess the influence of the rhizosphere on soil hydraulic properties. The effect of root hairs on P uptake has been analysed with image‐based models by Keyes *et al*. (2013) and Daly *et al*. (2016). These studies predict that, contrary to common past assumptions (Nye, 1966), root hairs contribute less or equal to P uptake than the root surface.

In this paper we present an imaging study in which we analyse root hair interactions with rhizosphere soil. The main goal of this study was to visualise and quantify soil structural changes induced by roots with distinct root hair morphology to document the impact of root hairs on soil structure. We tested two hypotheses: that root hairs influence the pore structure in the rhizosphere leading to a more structured soil; and that these changes are amplified by pore water fluctuations. To test these hypotheses we used the same hairless barley (*Hordeum vulgare* cv Optic) mutant studied by Haling *et al*. (2013) and Brown *et al*. (2013), alongside its wildtype parent. A root growth experiment contrasted these genotypes (*hairs* vs *no hairs*) using small growth microcosms that enabled high‐resolution SRCT imaging of root hairs and rhizosphere structure. We also used two contrasting water treatments, a wetting–drying cycle (*WD*) and a single drying treatment (*D*), on the wildtype plants to investigate the interactions between root hairs and soil water regime. Digital image analysis was used to document and quantify the interactions between root hairs and soil structure. As the link between structural and functional parameters remains a challenge, numerical models were applied to the imaged geometries to simulate water and solute movement in the rhizospheres of the contrasting genotypes. Our findings enhance our understanding of how rhizosphere formation is impacted by genotypic variations in root hair density (RHD), and how these changes affect fundamental plant uptake processes.

## Materials and Methods

### Plant growth and sample preparation

**Figure 1:**
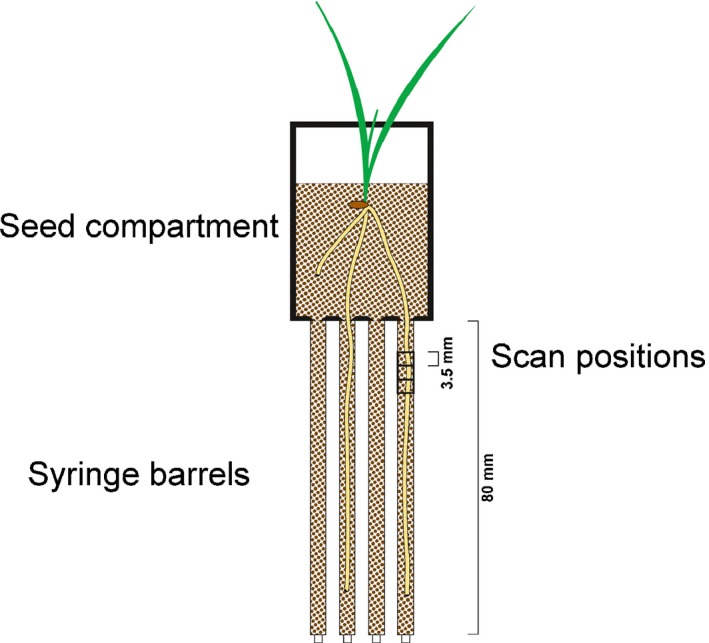
Schematic drawing of the root growth assembly used for barley roots in this study. The bottom of the seed compartment was designed to guide individual roots into syringe barrels. There were seven syringe barrels connected to each seed compartment.

Individual barley plants (*Hordeum vulgare* L. cv Optic) were grown in 3D printed seedling holder microcosms, first used by Keyes *et al*. (2013). A root‐hair‐bearing wildtype (henceforth referred to as *hairs*) and a plant line with greatly decreased root hair growth (*no hairs*) as described by Brown *et al*. (2012) were selected from the barley mutant population at The James Hutton Institute (Caldwell *et al*., 2004). Seeds were pregerminated on 1% distilled water agar for 48 h. Seven 1 ml syringe barrels (height = 80 mm, inner diameter = 4.2 mm) were inserted into a larger tube of 30 mm diameter, and filled with sandy loam textured soil (Dystric Cambisol, sieved to < 1 mm) to a density of 1.2 g cm^−3^. This soil was collected from the South Bullionfield at the James Hutton Institute. Syringe barrels were connected to the microcosms such that individual roots could grow into the syringe barrels (Fig. 1). A single barley seedling was planted in each assembly. Plants were grown in a glasshouse (at *c*. 20°C during the day) for 8 d before harvest. A preliminary experiment observed roots growing through the tip of the syringe after 10 d. Tubes were connected to the base of each syringe barrel, which were filled with water and connected to a reservoir that could be raised or lowered. A wetting/drying (volumetric water content θ *c*. 0.22–0.25 g g^−1^) treatment (*WD*) was applied by lifting the water table to saturation every 2 d and subsequently leaving samples to drain. An additional drying (*D*) treatment (θ *c*. 0.18 g g^−1^) was applied for the *hairs* genotype to explore the effect of hydrological stresses on structure development within the rhizosphere. In the drying treatment, plants were gently watered from the top with sufficient water to prevent desiccation, with the tube removed from the base of the syringe barrel. Plants were transported live to the synchrotron and, after harvest, individual syringe barrels were excised from the assemblies and sealed with Parafilm. A total of 34 replicate roots were imaged.

### Synchrotron radiation computed tomography

After plant growth, SRCT scanning was carried out at the I13 beamline at the Diamond Light Source, Oxfordshire, UK. Individual syringe barrels were scanned at three different heights (3.5 mm apart) starting near the upper end of the syringe barrel to maximise the chance of finding roots. This resulted in a total vertical extension of the scanned region of 10.5 mm, which ensured that the scanned roots had comparable age. SRCT was performed using ‘pink light’ at energies of *c*. 15–20 keV. In total, 1601 equiangular projections through 180° were recorded with an exposure time of 0.15 s per projection. The total duration of an individual scan was 4 min. X‐rays were scintillated using a 500 μm cadmium tungstate (CdWO_4_) scintillator, with a PCO edge 5.5 CMOS detector used to image the generated light. A microscope system with a four‐fold optical magnification was used, resulting in a field of view of 4 × 3.5 mm at 1.6 μm pixel size. The propagation distance was 63.5 mm, leading to an intermediate amount of phase contrast. Edge enhancement was estimated to be 20% of the dynamic range, which complicated soil segmentation, but improved the visibility of root hairs. Reconstruction of 3D images from the attenuation data was carried out with a filtered back‐projection algorithm and converted to stacks of 2160 slices each comprising 2560 × 2560 pixels with 32‐bit dynamic range.

### Image preprocessing

**Figure 2:**
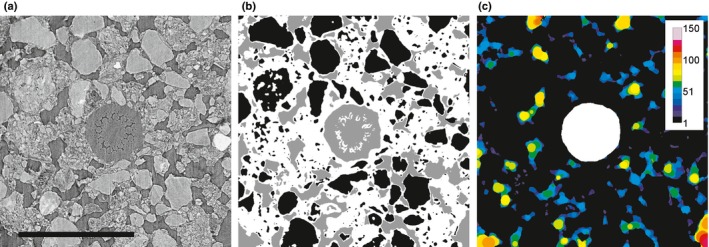
(a) Cross‐section of barley root (*no hairs*) growing in soil. Internal root structures and the surrounding soil structure could be clearly visualised. Bar, 1 mm. (b) Soil classification using trainable WEKA segmentation. Black, solid phase; white, mixed phase; grey, air‐filled pore space. Note that the root was segmented independently. (c) Pore size classification around the root. Segmented root is shown in white. Colours indicate local pore diameter in micrometres.

Image analysis was performed in ImageJ and Avizo 9.0.1 (FEI Visualization Sciences Group, Houston, TX, USA). The contrast was enhanced using histogram equalisation, and reconstructed images were then converted to 8 bit to reduce the computational cost of image analysis. Since not all 34 replicates produced results viable for further analysis, a set of criteria for sample selection was defined. Roots had to be closer to the centre of the syringe barrel than to the barrel wall to reduce edge effects. Scans containing major macropores (*n *=* *6) in the analysed region or more than one main root axis per syringe barrel (*n *=* *6) were removed. Additionally, shrunken and potentially desiccated roots (*n *=* *6) were removed. This reduced the number of useful images to five reps each for *no hairs WD* and *hairs D*, and four reps for *hairs WD*. In each viable image a smaller region of interest (ROI) of 2 × 2 × 1 mm with a root in the centre was cropped for further analysis (Fig. 2a). A rotational transformation was performed to ensure the root was in the centre along the entire ROI height.

### Segmentation

Roots and root hairs were segmented manually in Avizo 9.0.1 using a graphical tablet and scrolling through horizontal slices. Soil was segmented into three different phases (Fig. 2b): primary minerals (*Solid*), air‐filled pores (*Pore*), and a mixed phase comprising small, water‐filled pores and silt/clay‐sized solid particles below resolution (*Mixed*). A detailed description of the segmentation procedure is available as supporting information in the online version of this article (Supporting Information Methods S1, Fig. S1, Table S1).

### Quantification of structural parameters

Pore size distribution (PSD) was measured using the local thickness tool from the ‘Bonej’ plugin in ImageJ. The method generates a pore size map (Fig. 2c), where the grey value of each point of the pore space represents the diameter of the largest ball that fits entirely into the pore space and includes this point. PSD is given by the histogram of the resulting image. This definition of PSD is closely related to the hydraulic behaviour of pores (Vogel *et al*., 2010).

The Euclidean distance transform of the binary root image was generated and segmented into annuli (thickness = 50 μm) with increasing distance from the root surface. RHD was calculated by skeletonising the root hairs and measuring the skeleton length density within discrete annuli. Volume fractions of the distinct soil phases were calculated as the volume of the considered phase within an annulus divided by the total annulus volume.

For measurement of pore connectivity and image‐based modelling, *n *=* *20 cubic subvolumes of 500 μm side length were generated in each image. The size of the subvolumes was chosen based on convergence of simulated diffusion and permeability data (see the following section). Coordinates of the subvolumes were randomly selected with the constraint that the subvolume had to be outside the main root axis, and the maximum overlap between two subvolumes was 250 μm on any axis.

Pore connectivity was measured by labelling connected pore clusters (using the 18‐connected neighbourhood, that is, any pixel that touches one of the faces or edges of the original pixel) and calculating a dimensionless connectivity index (Renard & Allard, 2013)

(Eqn 1) $$\Gamma_{p}=\frac{1}{N_{p}^{2}}\sum_{i=1}^{N_{\iota }}v_{i}^{2},$$

where any cluster of the *pore* phase *p* has a volume *v*_*i*_, $N_{\iota }$ is the number of clusters and *N*_*p*_ is the total volume of the *pore* phase. For the calculation of $\Gamma_{p}$ the volume of each individual cluster and the total pore volume within each subvolume were determined and (Eqn 1) was solved. This was subsequently repeated for subsets of the pore space which included only pores of decreasing maximum diameter. This was done by thresholding the pore size map at incrementally reduced thresholds with steps of 10 μm. This procedure simulates a drying experiment and gives an estimation of pore connectivity at decreasing soil matric potentials.

To calculate the percolation threshold, that is, the pore size at which the pore clusters become disconnected, a logistic equation was fitted to the data

(Eqn 2) $$\Gamma_{pf}\left(d\right)=\frac{\Gamma_{p,\max}}{1+e^{-a(d-d_{0})}},$$

where $\Gamma_{p,\max}$ is the connectivity of the entire pore space, *d* is maximum pore diameter and *d*_0_ is the maximum pore diameter at the percolation threshold, and *a* is a fitting parameter.

### Image‐based modelling of effective diffusion and permeability

For the image‐based modelling the same set of subvolumes created for the connectivity measurement was used. For each subvolume an STL surface mesh was generated using ScanIP (Simpleware Ltd, Exeter, UK). We used saturated conditions, that is, *pore* and *mixed* phases were combined to produce the fluid phase. For every subvolume, seven smaller test volumes of different sizes were generated. This was done to ensure that the final subvolumes were representative elementary volumes (REVs), that is, their pore geometry is representative of the pore geometry of the entire sample. These were numbered 0–6. The side length of the test volumes can be calculated using

(Eqn 3) $$L^{3}=\frac{L_{0}^{3}}{2^{i}},$$

where *L* is the test volume side length, *L*_0_ is the original subvolume side length and *i* is the test volume number (*i *=* *0 corresponds to the largest and *i *=* *6 corresponds to the smallest).

For each test volume, separate simulations were carried out to measure the impedance to solute diffusion and the hydraulic permeability in the *x*,* y* and *z* directions, respectively. Impedance to diffusion presented by the soil was calculated in terms of an effective diffusion constant *D*_eff_ from the soil geometry using the method described in detail by Daly *et al*. (2016).

If the subvolume qualifies as an REV, solute diffusion in the soil is thus described by:

(Eqn 4) $$\frac{\partial C}{\partial t}=\nabla \left(DD_{\mathrm{eff}}\nabla C\right),$$

where *D* is diffusion constant in pure water, and *C* is solute concentration.

Likewise, the hydraulic permeability *k* offered by soil geometry was calculated. The detailed method is described by Tracy *et al*. (2015). Given an external fluid pressure gradient, the resulting velocity is

(Eqn 5) $$u=-\frac{k}{\eta }\left(\nabla p-\rho g{\hat{e}}_{z}\right),$$

where η is the viscosity of the fluid, *p* is the applied pressure, ρ is the density of the fluid, *g *=* *9.8 ms^−2^ is the acceleration due to gravity, and ${\hat{e}}_{z}$ is the unit vector in the vertical direction. Numerical simulations (*n *=* *5880 for each *k* and *D*_eff_) were carried out using OpenFOAM, an open source fluid dynamics toolbox on IRIDIS, the High Performance Computing Facility at the University of Southampton. *D*_eff_ and *k* are soil properties; however, if the domain is too small to qualify as an REV they are also a function of the domain size. To overcome this, *k* and *D*_eff_ were fitted with the functions

(Eqn 6) $$D_{\mathrm{eff}}=a+be^{-cL},\;\text{or}\;k=a+be^{-cL},$$

where *a*,* b* and *c* are fitting parameters and *L* is the side length of the domain. The fitted diffusion coefficient is the limit of this equation as *L* tends to infinity, that is, *D*_eff_ = *a*.

Statistical analysis was carried out in Matlab 2015a (The MathWorks Inc., Natick, MA, USA). We used ANOVA for normally distributed variables and Kruskal–Wallis test for nonparametric data. For pairwise *post hoc* comparisons, the Dunn–Bonferroni approach was used.

## Results

### Overall plant performance

The *no hairs* genotype had a significantly greater fresh shoot mass and plant height, while the drying treatment had no significant effect on shoot mass or plant height (Table 1). Where roots had grown into syringe barrels, they generally extended along the entire length of the barrels (8 cm), but in some cases roots escaped the lower end of the barrels.

**Table 1:** Measured parameters of barley plants

	Plant height (cm)	Fresh shoot mass (g)	No. of roots analysed	Root diameter (mm)
*Hairs WD*	7.3 ± 2.1	64.5 ± 25.2	4	0.47 ± 0.02
*Hairs D*	8.0 ± 0.6	65.3 ± 11.1	5	0.47 ± 0.03
*No hairs WD*	10.4 ± 1.3*	107.3 ± 23.9*	5	0.49 ± 0.02

Data are mean ± SD. Asterisks denote significant differences between treatments (*P *<* *0.05). *WD*, wet–dry treatment; *D*, dry treatment.

In the SRCT images roots could be clearly distinguished from soil, including root internal structure, comprising intercellular and aerenchymous spaces. Root diameter, obtained by measuring the area of the segmented root in each slice and assuming a cylindrical shape, showed no significant difference between genotypes (Table 1).

### Root hair density

**Figure 3:**
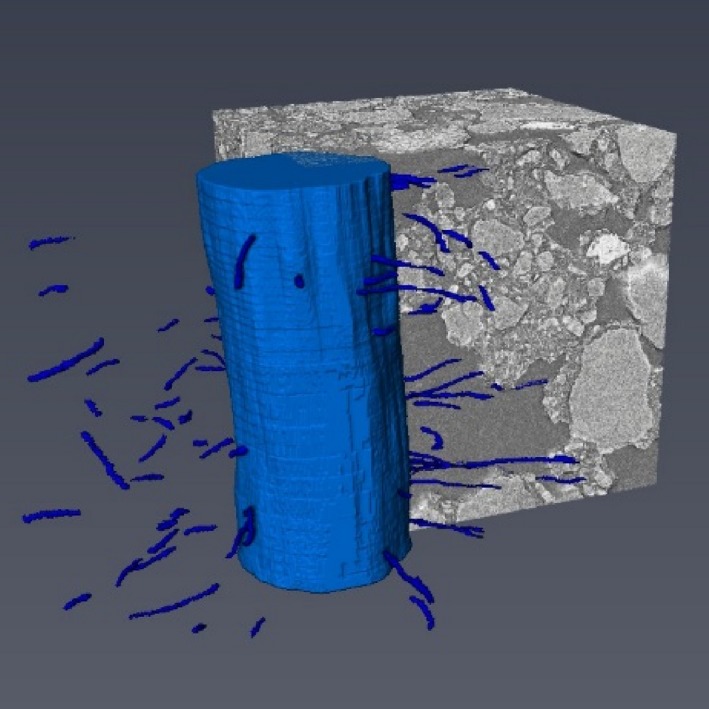
3D rendered barley root (*hairs WD*) and hairs including a region of interest showing the surrounding soil. Light blue structure is the segmented root, while dark blue structures are segmented root hairs within a region of interest (ROI) of 2 × 2 × 1 mm. Vertical length of the root is 1 mm. Only root hairs growing in air‐filled pores could be seen, and hence root hair structures are fragmented.

**Figure 4:**
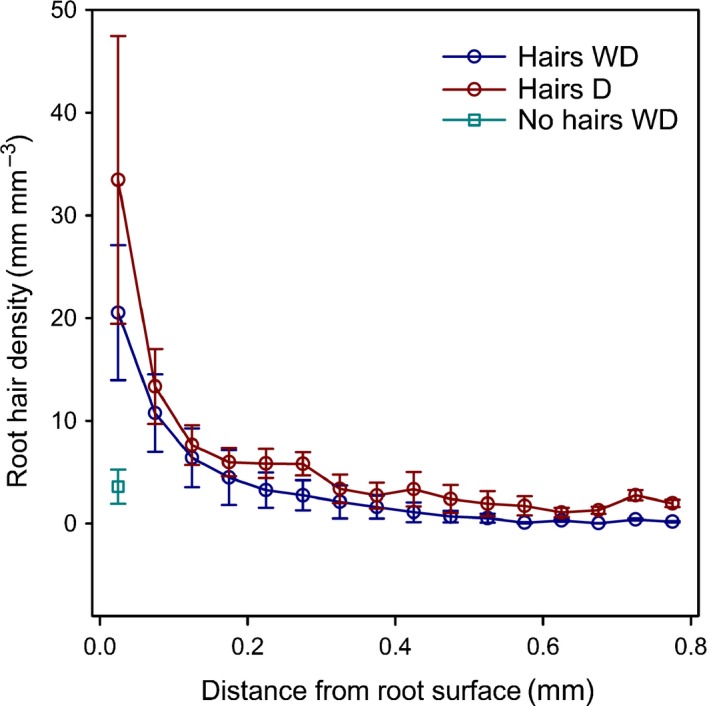
Mean root hair density of barley roots over distance from the root surface. Each value represents mean root hair density within an annulus of thickness 0.05 mm about the root centre. The *x* values represent the inner diameter + 0.025 mm of each annulus. Error bars represent ± SE of the mean. The *no hairs* genotype only had short hairs in the innermost annulus. *WD*, wet–dry treatment; *D*, dry treatment.

Root hairs were clearly visualised in air‐filled pores, but they were more difficult to detect within the mixed phase. To avoid error induced by subjective user interpretation, only clearly visible root hairs were segmented. Some of the resulting root hair structures were fragmented and disconnected (Fig. 3), indicating that root hairs grew into both the air‐filled *pore* phase and the *mixed* phase. The average number of root hairs counted at the immediate root surface along a 1 mm root segment (derived from counting discrete skeletons) was 24, ranging from 0 in the *no hairs* genotype to 60 in the *hairs* genotype and *D* treatment. The resulting mean RHDs at the immediate root surface were highly variable, ranging from 5.4 to 94.2 mm mm^−3^ in the *hairs* genotype. RHD decreased exponentially with distance from the root surface (Fig. 4) and was not significantly different between *D* and *WD* treatments. To explain the larger variability of RHD close to the root, we calculated the correlation coefficient between RHD and pore volume fraction within each distance class. RHD was significantly correlated with pore volume fraction within the 0.3 mm volume closest to the root (Pearson's *r *>* *0.7, *P *<* *0.05), but further away from the root no correlation between hair density and pore volume was found.

The *no hairs* genotypes bore short root hair stumps, which only grew within the innermost 0.05 mm from the root surface. Further away from the root no hairs were found for the *no‐hairs* genotype. RHD within the innermost annulus was 3.6 mm mm^−3^ for *no hairs*, which was significantly less than for *hairs D* (*P *<* *0.05) but not *hairs WD* (probably due to the large variability in hair length density within this narrow zone).

### Soil structure

**Figure 5:**
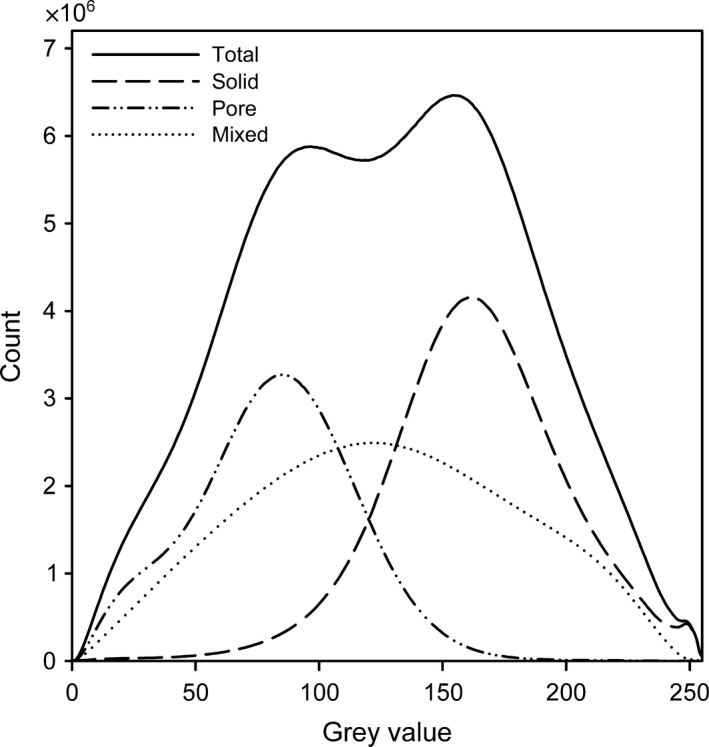
Grey value histograms of the total 3D region of interest in the barley rhizosphere and the different segmented phases showing overlapping grey values of the different phases, particularly the *mixed* phase.

The soil segmentation resulted in images consisting of three phases: the *pore* phase, consisting of air‐filled pores ≥ 5 μm, a *mixed* phase consisting of smaller water‐filled pores and solid particles of the silt and clay fractions, and a *solid* phase consisting of larger particles with undetectable internal porosity. As previously noted, the segmentation results showed a slight overestimation of the mixed phase caused by partial volume effects. There was substantial overlap of the grey values of the different phases (Fig. 5), which was intensified by the edge enhancement due to phase contrast. This was especially true for the *mixed* phase, which had a large impact from edges that causes a broad grey‐value histogram.

**Figure 6:**
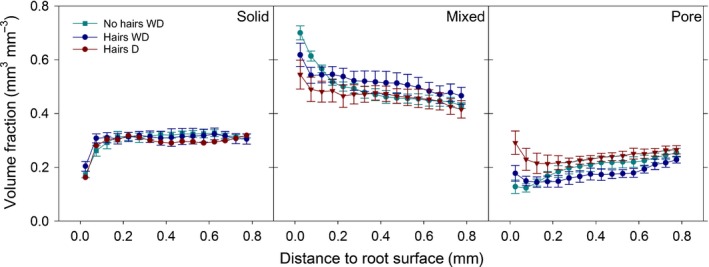
Volume fractions of *solid*,* mixed* and *pore* phase, respectively, over distance from barley root surface. Data are mean volume fractions within an annulus of 0.05 mm diameter; *x*‐values are annulus inner diameter + 0.025 mm. Error bars represent ± SE of the mean. *WD*, wet–dry treatment; *D*, dry treatment.

Volume fractions of the different phases were analysed with distance from the root surface to quantify the impact of root activity on soil structure (Fig. 6). *Solid* volume fraction was uniform across the ROI, but sharply decreased close to the root surface, although the effect of distance was only significant for *no hairs* and *hairs D* (*P *<* *0.05). There was no significant difference in *solid* volume between treatments.

The *mixed* phase showed an increased volume fraction close to the root surface for all treatments. In the *no hairs* genotype this increase was larger and extended further away from the root surface. The effect of distance on mixed phase volume fraction was consequently only significant for *no hairs* (*P *<* *0.05). Comparison of treatments showed that *hairs D* had a significantly smaller *mixed* volume fraction than the other two treatments (*P *<* *0.05).

The *pore* volume fraction decreased significantly with distance from the root for *no hairs* and *hairs WD* (*P *<* *0.05). Pairwise comparisons of individual annuli showed no significant differences in *hairs WD*, while in the *no hairs* genotype the *pore* volume fraction in annuli at the root interface (from 0.05 to 0.15 mm) was significantly smaller than in the most distant annuli (0.8–1 mm). There was no significant change in *pore* volume fraction with distance for *hairs D*. Comparison of the treatments showed that all treatments had significantly different *pore* volume fractions (*P *<* *0.05). Overall *pore* volume fraction was greatest in *hairs D* and smallest in *hairs WD*.

### Pore size distribution

**Figure 7:**
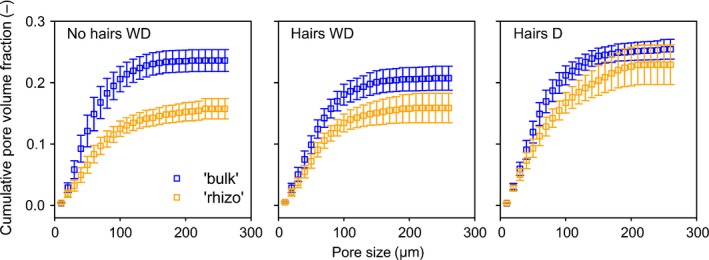
Cumulative pore size distribution at different distances from the barley root surface. ‘Bulk’, pore size distribution at 500–800 μm from the root surface; ‘Rhizo’, pore size distribution at 0–300 μm from the root surface. Only pores > 5 μm were characterised. *WD*, wet–dry treatment; *D*, dry treatment. Error bars represent ± SE of the mean.

Due to limitations of resolution we did not estimate porosity *per se*, but cumulative PSD for the *pore* phase was calculated from the pore size map. To analyse the effect of distance from the root, the closest annuli within 0.3 mm distance from the root (‘rhizo’, Fig. 7) were grouped and compared to annuli from 0.5 to 0.8 mm distance (‘bulk’, Fig. 7). The results confirm the smaller pore space (> 5 μm; i.e. localised compaction) around the roots of the *no hairs* genotype compared to the *hairs* genotype. To analyse pore size distribution independent of the total pore volume, PSD was normalised to the total pore volume within each annulus at different distances from the root. The resulting normalised distributions were compared for statistical differences with a two‐sample Kolmogorov–Smirnov test. Normalised PSD was not significantly different between ‘rhizo’ and ‘bulk’, nor between different treatments. However, we document the trend of normalised PSD over distance from the root in Fig. S2. In *no hairs* normalised PSD was slightly wider close to the root surface, with a greater frequency of bigger pores. The bulk of the distribution was unchanged. In *no hairs WD* the opposite trend was observed; normalised PSD became narrower close to the root surface, but again the bulk of the distribution was fairly constant over distance. In *hairs D* the overall widest normalised PSD and the most significant change over distance was observed. Normalised PSD was notably wider close to the root surface.

### Pore connectivity

Pore connectivity was estimated in randomised subvolumes distributed across the entire ROI. Total pore connectivity $\Gamma_{p}$ was greatest in the *hairs D* treatment and least in *hairs WD* (Table 2)*. No hairs WD* had an intermediate $\Gamma_{p}$. Treatment effects were significant, and pairwise comparison showed that only *hairs WD* had significantly different $\Gamma_{p}$ from the other treatments. $\Gamma_{p}$ correlated significantly with the pore volume fraction of the subvolumes (Pearson's *r *=* *0.77, *P *<* *0.05). The percolation threshold (i.e. the pore size at which the pore clusters become disconnected) was lowest in *hairs D*, intermediate in *no hairs WD* and highest in *hairs D*. Treatment effects were significant; pairwise comparison showed that the *hairs D* was significantly different from the other treatments. There was no correlation between percolation threshold and $\Gamma_{p}$ (*r *=* *0.16). The two results combined show that connectivity was greatest in *hairs D* and was maintained longer when the large pore bodies were removed. Conversely, overall connectivity was least in *hairs WD,* which coincided with an earlier breakdown of connectivity when removing large pore bodies.

**Table 2:** Connectivity parameters and simulation results of barley rhizosphere

Treatment	$\Gamma_{p}$ total	Percolation threshold (μm pore size)	Pore volume fraction (saturated pore volume fraction) (−)	Simulated *D*_eff_ (−)	Simulated *k* (× 10^−6^ cm^2^)
*No hairs WD*	0.81 ± 0.23	53 ± 13	0.22 ± 0.07 (0.69 ± 0.08)	0.78 ± 0.12	1.48 ± 0.83
*Hairs WD*	0.73 ± 0.24*	55 ± 14	0.19 ± 0.07 (0.70 ± 0.06)	0.80 ± 0.07	1.47 ± 0.95
*Hairs D*	0.86 ± 0.21	48 ± 14*	0.23 ± 0.07 (0.71 ± 0.08)	0.80 ± 0.06	1.60 ± 0.99

Data are mean ± SD in *n *=* *100 subvolumes (80 in the case of *hairs WD*) per treatment. *D*_eff_ is the average relative effective diffusion coefficient in fully saturated soil, *k* is average saturated permeability and $\Gamma_{p}$ is the dimensionless connectivity index described in (Eqn 1). Asterisks denote statistical differences between treatments. *WD*, wet–dry treatment; *D*, dry treatment. Size of the individual subvolumes was 500 × 500 × 500 mm.

### Simulation results

**Figure 8:**
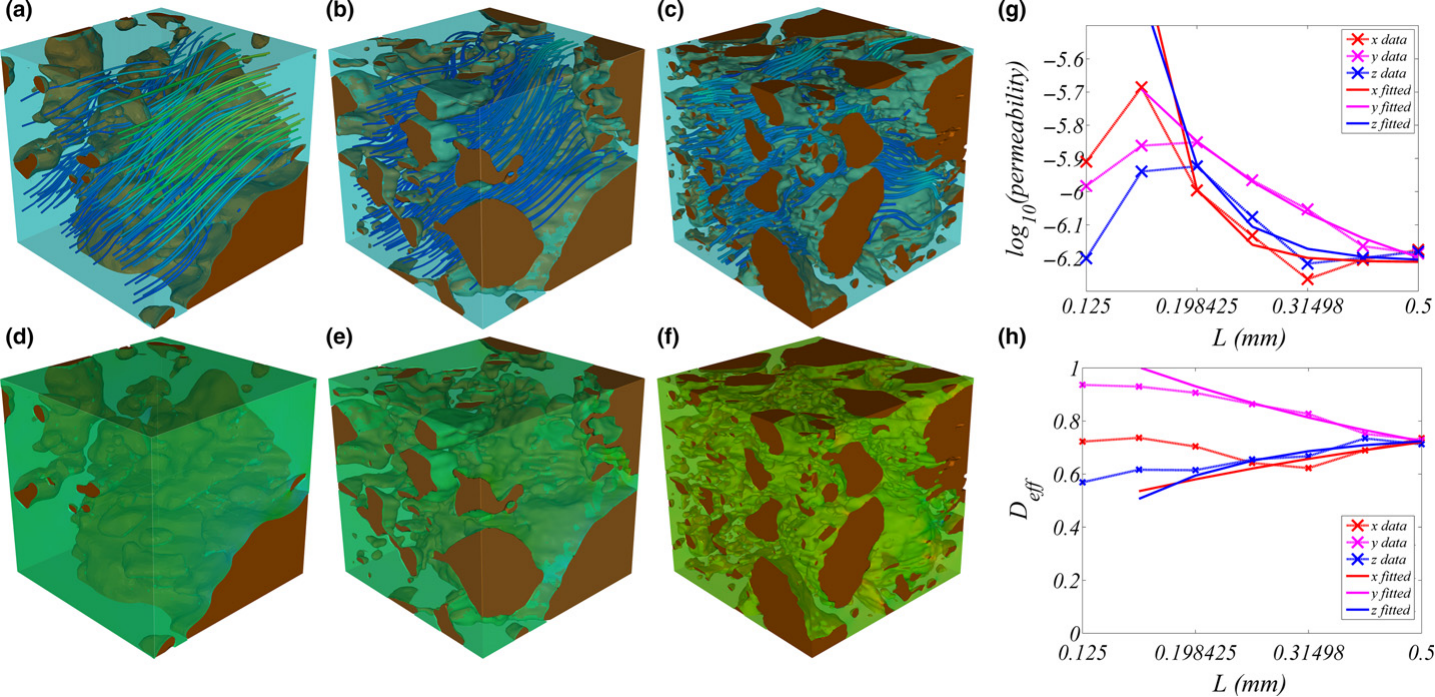
Image‐based modelling of relative permeability *k* and effective diffusion constant *D*_eff_ in the barley rhizosphere. (a–f) Simulation results for selected test volumes of increasing side length (left: 0.16 mm; middle: 0.25 mm; right: 0.4 mm) taken from a sample of *hairs WD*. Note that the smaller test volumes are subsets of the larger ones. (a–c) Flow streamlines show local Darcy velocities. Warmer colours indicate greater relative velocity. (d–f) Colours show relative impedance to diffusion. (g, h) The convergence of (g) *k* and (h) *D*_*eff*_ in the *x*,* y* and *z* directions with increasing test volume size. Dashed lines show simulated values and solid lines the exponential fit.

Fig. 8 shows typical distributions of permeability and effective diffusion constants within the imaged geometries. Convergence of *k* and *D*_eff_ was typically achieved at a subvolume side length of 500 μm (Fig. 8g,h). This was, however, not the case for all the subvolumes, where either the exponent *c* in the fitted exponential equation (Eqn 6) was too small, meaning that no convergence was achieved, or the quality of the fit was insufficient. We therefore applied thresholds on both the exponent (*c *>* *0.5) and the quality of the fit (root mean square error (RMSE) < 0.05) to exclude outliers. Removal of outliers did not significantly alter the saturated pore volume fraction. The resulting *D*_eff_ was calculated in the *x*,* y* and *z* directions. Interestingly, ANOVA showed that *D*_eff_ was significantly less in the *z* direction (*P *<* *0.05), but did not differ in the *x* and *y* directions. The averaged *D*_eff_ was similar in all treatments and no statistically significant difference was observed (Table 2). Likewise, there was no significant effect of the distance of subvolume centroids from the root surface. However, *D*_eff_ correlated with saturated pore volume fraction of the subvolumes across all treatments (*r *=* *0.77, *P *<* *0.05).

Simulation results for permeability (*k*) were analysed in the same way as the diffusion results. For the removal of outliers, thresholds on the exponent (*c *>* *0.5) and the goodness of the fit (RMSE < 0.5) were used. The resulting *k* showed no statistically significant differences between the *x*,* y* and *z* directions. Similar to the simulated diffusion, there were no statistically significant differences between treatments, or over distance of subvolume centroids. *k* correlated with saturated pore volume fraction of the subvolumes (*r *=* *0.57, *P *<* *0.05). However, the correlation was smaller than the correlation between *D*_eff_ and saturated pore volume fraction.

## Discussion

### Root hair impact on soil structure

Root hairs had a significant effect on soil structure formation in the rhizosphere. Root hairs were shown to influence porosity and connectivity for the ≥ 5 μm pores visualised with SRCT. Hydrological stress history, imparted as drying only, or a cycle of wetting and drying, also had a large impact on the developed pore structure.

**Figure 9:**
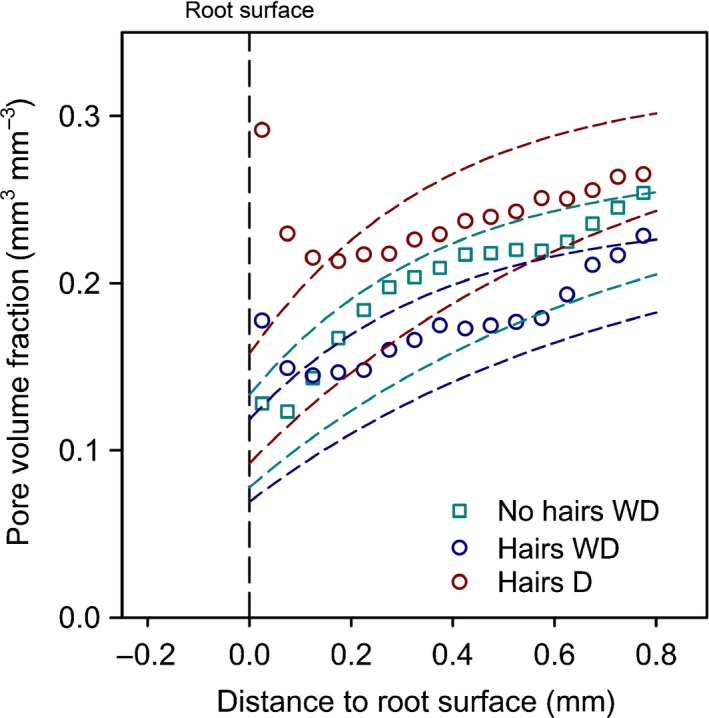
Predicted pore volume fraction over distance from the barley root using the model of Dexter (1987). Dashed lines show the predicted pore volume fraction for each sample. Mean pore volume fraction at 1 mm distance was used as the bulk porosity for each treatment. Upper and lower dashed lines of matching colours show the predicted pore volume fraction using soil mechanical parameters *k*_D_ = 0.68 and 0.34, respectively. Data points show mean pore volume fractions obtained from image analysis. *WD*, wet–dry treatment; *D*, dry treatment.

Whilst all treatments showed evidence of soil compaction gradients around the roots, estimated by the increased volume fraction of the fine textured *mixed* phase, the *hairs* genotype had a greater pore volume close to the root soil interface compared to *no hairs*. Using the exponential model for soil deformation around roots proposed by Dexter (1987), we calculated the expected decrease in porosity due to root expansion. We used the pore volume fraction measured in the most distant annulus of soil as bulk porosity and calculated the root radius from the segmented root volume assuming a cylindrical shape. For soil mechanical parameter *k*_D_, we used the values for different remoulded soils given by Dexter (1987). The results show that the reduction of pore volume for the *no hairs* genotype could be described by Dexter's model (Fig. 9). Interestingly, in the *hairs* genotype the measured pore volume far away from the root was described well by Dexter's model. However, near to the root surface the pore volume fraction deviated significantly from this model. This indicates that the initial compression of soil around the growing root tip was similar for all treatments and the impact of root hairs was to locally disrupt the porosity close to the root surface. This hypothesis is supported by the similar distribution of the incompressible *solid* sand fraction around the roots. While sand displacement should theoretically lead to an increased fraction of particles close to roots, this was not observed in our study. In the annulus closest to the root surface the *solid* sand fraction decreased sharply, probably as a consequence of the packing geometry of particles along the curved root surface. Our results show that root hairs increased the (> 5 μm) pore volume at the root–soil interface within a zone of *c*. 200 μm distance from the root. This localised effect was amplified further in the drying only treatment. This raises the question of whether root shrinkage may have caused the formation of air gaps between roots and soil; Carminati & Vetterlein (2013) showed this to be important for lupin in drought conditions. The occurrence of gaps in our experiment is unlikely since air gaps in Carminati & Vetterlein (2013) appear after prolonged drought conditions not present in our study. Note that we did not measure porosity *per se*, as the imaging resolution did not permit the identification of pores < 5 μm.

Soil porosity is often divided into a textural and a structural component, where the textural component is determined by the distribution of primary soil minerals, and the remaining porosity is the structural component (Nimmo, 1997). In our study, the air‐filled *pore* phase is roughly identical to the structural component. The volume of the structural component is expected to decrease upon soil compression (Kutílek *et al*., 2006), which matches our observation in the *no hairs* genotype. However, in the *hairs* genotype we observed a secondary increase in detectable pore structure, signifying a shift from smaller to larger pores. Interestingly, pore size distributions were fairly stable and did not show an obvious pattern for either treatment. Upon compression, the fraction of large pores is expected to decrease, but in *no hairs* a decrease of the largest pore fraction was only observed within 200 μm distance from the root surface, where a local maximum was observed. Further away from the root the fraction of larger pores decreased again, which is counterintuitive. Note that the initial soil conditions were fairly heterogeneous, as evidenced by the large variability of pore size distribution, which may explain this observation. In the *hairs* genotype a similar pattern was observed for the *WD* treatment only, which indicates an impact of the multiple drying and rewetting cycles. The frequency of smaller structural pores is expected to increase with each drying cycle at the expense of larger pores (Leij *et al*., 2002). Drying cycles will be more severe close to the root surface, and hence this effect could only be observed close to the root.

The results suggest greater pore structure formation away from the root for the plants with root hairs. It is likely that this is driven by the expansion of the hydraulic gradient from the root surface due to root hair activity, as suggested by Segal *et al*. (2008). Figs 7 and 9 demonstrate the combined importance of the hydraulic stress and root hairs on the development of pore structure. Many studies have demonstrated the importance of wetting–drying cycles, and the presence of biological exudates, to soil structure formation (Peng *et al*., 2011). Direct physical rearrangement of soil particles by growing root hairs is another plausible mechanism, as it has been shown that root hairs are able to deform moderately resistant clays (Champion & Barley, 1969) and are able to transmit tensile forces between root and soil (Bengough *et al*., 2016).

While structural differences between the *hairs* and *no hairs* genotypes were generally confined to a volume of *c*. 200 μm diameter around the root, we observed significant differences in the overall connectivity of the *pore* phase between the genotypes. However, connectivity is a function of pore size (Vogel, 1997) and the differences observed in this study were mostly explained by differences in pore volume fraction of the measured subvolumes. The biggest differences were observed between the different wetting treatments. The percolation threshold was unaffected by the genotype but was significantly smaller in the drying‐only treatment, which indicates a higher pore‐neck connectivity. Both results emphasize the impact of hydraulic drivers on pore structure.

### Image‐based modelling

Simulation results showed that the effective saturated diffusion and permeability were unaffected by both genotype and water treatment. Likewise, the centroid distance of the subvolumes from the root surface had no significant effect on both *D*_eff_ and *k*. The subvolume size which qualified as an REV was *c*. 500 μm. This was too large to measure the effect of distance to the root surface. Since diffusion and permeability were simulated in saturated conditions, no significant differences were to be expected, because the combined *pore* and *mixed* fractions were unaffected by the treatment. However, both *D*_eff_ and *k* correlated with saturated pore volume fraction, which allowed their behaviour to be predicted in unsaturated conditions, that is, when water and solute flow are constrained to the *mixed* phase. Assuming that the unresolved internal porosity within the *mixed* phase was similar between treatments, the resistance to water and solute flow should be related to the volume fraction of the mixed phase, which was greater close to roots in the *no hairs* genotype. This suggests that root hairs may decrease unsaturated hydraulic conductivity and solute diffusivity in the rhizosphere compared to hairless genotypes. Although this suggests that both water and nutrient uptake by the root would be impeded in unsaturated conditions in the hairs genotype, uptake by hairs might counteract this impact. The role of root hairs in resource capture remains poorly understood, although Segal *et al*. (2008) found that no hairs mutants were less effective at drying rhizosphere soil. Even if root hairs do not take up water directly, they may provide film flow pathways for water by bridging air‐filled pores. While previous image‐based modelling studies showed that greater inter‐aggregate contacts caused by root‐induced compaction allow plants to extract more water from the soil (Aravena *et al*., 2011, 2014) we show that root hairs may significantly alter this effect.

### Root hair quantification

In agreement with Keyes *et al*. (2013) we show that SRCT is appropriate to visualise how pore morphology is affected by root hair–soil interactions. However, there are some limitations. Root hairs were clearly visible within air‐filled pores, but when they were growing along soil minerals or within the *mixed* phase they were rendered invisible due to the smaller contrast to the surrounding medium. This is an important limitation, which leads to an underestimation of RHD. This may potentially be overcome by increasing propagation distance between scintillator and detector to increase edge enhancement or by using simultaneous phase and amplitude extraction algorithms (Paganin *et al*., 2002). The observed RHDs were less than the numbers reported for rice roots (Daly *et al*., 2016), which may be related to species differences or to the open textured growth medium that these authors used. We clearly show that RHD correlated with air‐filled pore volume within 300 μm from the root surface, which can indicate both a lower detection rate and a smaller actual RHD. While no significant difference in hair density was found between the *D* and *WD* treatments, we note that the detection rate of root hairs may be lower in *hairs WD* as a consequence of the lower pore volume fraction at the surface compared with *hairs D*. On the other hand, undetected root hairs may potentially increase the volume fraction of the *mixed* soil phase and consequently decrease the *pore* phase. Given the small volume of root hairs, the effect would be small compared to the observed differences in *pore* volume. Assuming a low hair detection rate of 10%, average RHD at the immediate soil–root interface would be 270 mm mm^−3^, which would translate to a difference in *pore* volume fraction of 1.4% for hairs of 8 μm diameter.

The fragmentation of the visualised root hairs clearly shows that they grew in both the air‐filled *pore* phase and the *mixed* phase, with transitions between these phases. Notwithstanding the limitations, comparison with destructive root hair measurements allows an estimation of the fraction of root hairs growing in air‐filled pores. Light microscope measurements of RHD in different barley lines have shown densities of up to 240 hairs mm^−1^ (Haling *et al*., 2010), which is an order of magnitude higher than the average measured in this study (24 hairs mm^−1^). This suggests that the majority of hairs are found within the fine textured mixed phase. Additionally, root hair counts were based on skeletonisation, which is unable to distinguish root hairs that are entangled. However, Daly *et al*. (2016) reported that RHDs measured in SRCT images were greater than those found in destructive analysis. Clearly, direct comparisons of SRCT images and microscope measurements of the same root sections are needed to confirm this. We found root hairs at distances of up to 800 μm away from the root, which was the maximum distance we analysed. This is not surprising, as previous work with the same genotype had determined that average root hair length was *c*. 800 μm in similar soil conditions (Brown *et al*., 2012). The absence of root hairs at greater distances than 50 μm from the root surface in the *no hairs* genotype confirms that the structures we found were indeed root hairs and not fungal hyphae, which can have similar size and shape.

In conclusion, the present study confirms that SRCT is a suitable technique to visualise root hair interactions with soil. The technique offers sufficient contrast and resolution to segment soil and root structures, including root hairs that grow in air‐filled pores. However, hairs growing in fine‐textured regions are not readily detectable. We showed that root hairs can counteract the effect of root‐induced soil compaction by significantly increasing pore volume fraction at the root–soil interface. Image‐based modelling predicted that these alterations would not significantly affect diffusion and hydraulic conductivity under saturated conditions, and are therefore estimated to have negligible impact on root water and solute uptake. However, it is likely that the *mixed* phase containing fine pores will have a substantial effect on transport into the root under a wide range of unsaturated conditions. The present study focused on local changes within short segments of roots at the same soil depth with comparable developmental stage. Changes of rhizosphere structure over root length or age and comparing roots of different diameters were beyond the scope of this work. As part of our research programme our follow‐on studies focus on the dynamics of rhizosphere formation.

## Author contributions

N.K., S.D.K., P.D.H., T.S.G., A.G.B. and T.R. designed the study. S.D.K., A.R., P.D.H., A.G.B., T.S.G. and L.K.B. collected data. N.K. analysed and interpreted the data. K.R.D. conducted the modelling. P.D.H. was the lead applicant of the BBSRC funding and synchrotron session. N.K. drafted the manuscript. N.K. drafted the manuscript. N.K., K.R.D., S.D.K., T.S.G., L.K.B., A.R., L.J.C., M.N., A.G.B., I.S., P.D.H. and T.R. conducted the critical revision and approval of the publication.

## Supporting information

Please note: Wiley Blackwell are not responsible for the content or functionality of any Supporting Information supplied by the authors. Any queries (other than missing material) should be directed to the *New Phytologist* Central Office.

**Fig. S1** Assessment of the WEKA segmentation process.
**Fig. S2** Normalised cumulative pore size distribution (PSD) over distance from the root surface.
**Table S1** Performance of iterative WEKA segmentation process
**Methods S1** Segmentation procedure.
