# Supplementary material for: High‐resolution synchrotron imaging shows that root hairs influence rhizosphere soil structure formation
Source: New Phytol. 2017 Jul 31;216(1):124–35. doi: 10.1111/nph.14705 (PMC5601222; doi:10.1111/nph.14705)
Supplement: Supplementary file 1 — Fig. S1 Assessment of the WEKA segmentation process. Fig. S2 Normalised cumulative pore size distribution (PSD) over distance from the root surface. Table S1 Performance of iterative WEKA segmentation process Methods S1 Segmentation procedure. [file NPH-216-124-s001.pdf]

***New Phytologist* Supporting Information**

Article title: High-resolution synchrotron imaging shows that root hairs influence rhizosphere soil structure formation
Authors: N. Koebernick, K.R. Daly, S.D. Keyes, T.S. George, L.K. Brown, A. Raffan, L.J. Cooper, M. Naveed, A.G. Bengough, I. Sinclair, P.D. Hallett and T. Roose
Article acceptance date: 12 June 2017

The following Supporting Information is available for this article:

Fig. S1 Assessment of WEKA segmentation process

Table S1 Performance of iterative WEKA segmentation process

Methods S1 Segmentation procedure

Fig. S2 Normalised cumulative pore size distribution (PSD) over distance from the root surface.

Fig. S1 Assessment of WEKA segmentation in a selected horizontal test slice of 480 x 480 micrometres. a) raw image, b) manual segmentation of *pore* phase and selected *solid* minerals used as ground truth data, c)-f) 1st – 4th iteration of the WEKA segmentation performed in the test slice, g) majority filter applied on 4th iteration, h) test slice taken from actual volume segmentation used for analysis, i) Otsu's thresholding algorithm (2 phases), j) open-close operation applied on Otsu segmentation. See Table S1 for performance measurement.

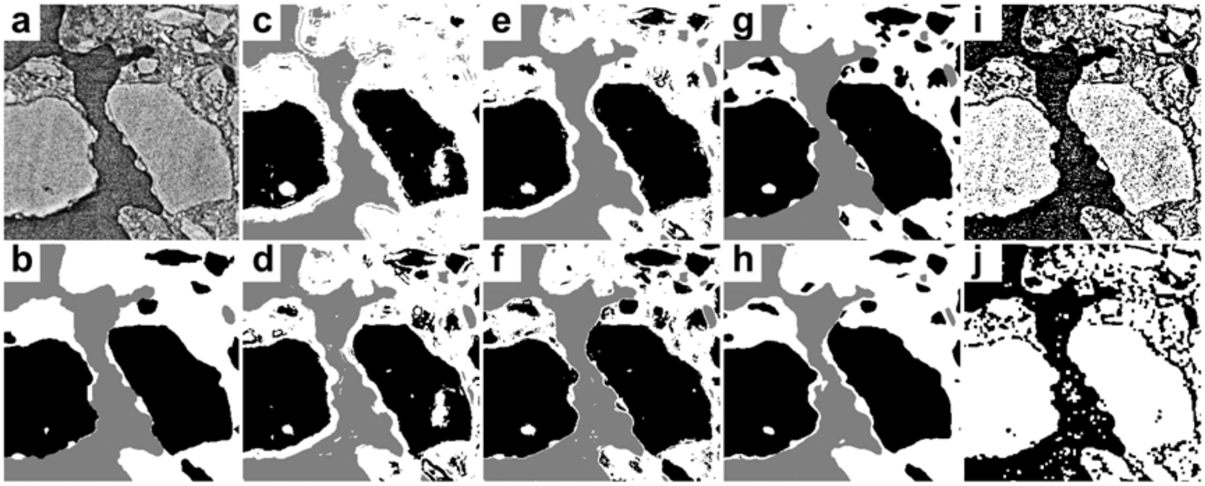

Fig. S2 Normalised cumulative pore size distribution (PSD) over distance from the root surface. Cumulative PSD was calculated within annuli of 0.05 mm thickness about the root for pore size increments of 10 µm. Cumulative PSD was normalised to the total pore volume within each annulus. The contour lines represent the percentiles of the PSD. A value of 0.5 means that 50% of the pore volume at distance x is made up by pores smaller than or equal to pore size y.

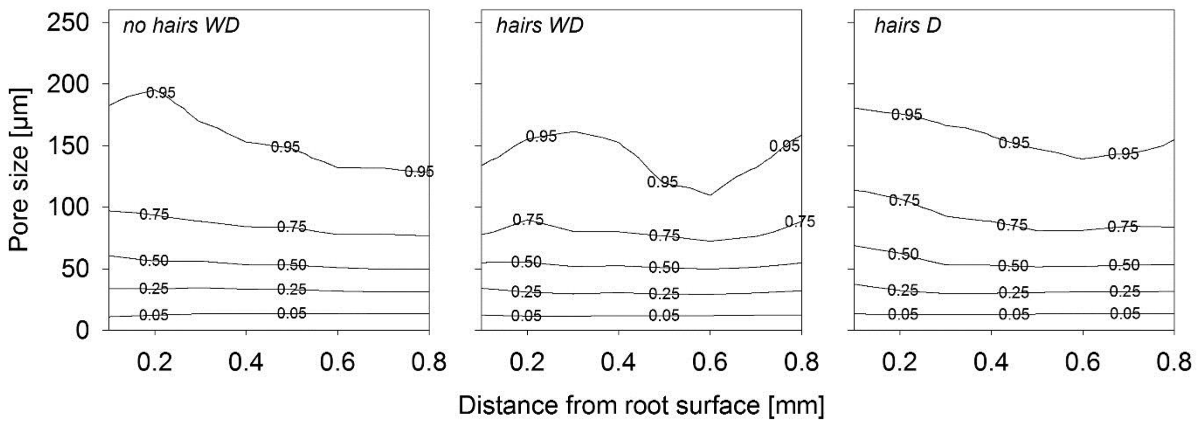

Table S1 Performance of iterative WEKA segmentation process in a representative test slice. Full volume WEKA segmentation (shown in bold) is the same slice taken from the full volume reconstruction used in this paper. For comparison, the test slice was segmented using Otsu's thresholding method and a smoothing operation (Note that this method only segments two phases). Numbers are the fractions of correctly classified pixels using manually segmented structures as ground truthing data.

Segmentation method	True classification *pore* phase	True classification *solid* phase
WEKA it. 1	0.77	0.69
WEKA it. 2	0.94	0.85
WEKA it. 3	0.96	0.84
WEKA it. 4	0.95	0.97
WEKA it. 4 + majority filter	0.96	0.97
**Full volume WEKA segmentation**	**0.90**	**0.93**
Otsu (2 phases)	0.91	0.90
Otsu (2 phases)+ open + close	0.93	0.99

Methods S1 Segmentation procedure for root and soil segmentation

Root segmentation

To classify the main root, a manual root cross-section was drawn every 50 slices using a brush tool and the intervening slices filled in via interpolation. Root hairs were tracked and marked manually using a brush tool while scrolling through the image until no further root hairs were found. To reduce user bias only root hairs that could be clearly distinguished from the background were included, i.e. the root hairs growing in the air-filled pore space.

Soil segmentation

Soil was segmented into three different phases: Primary minerals (*Solid*), air-filled pores (*Pore*), and a mixed phase comprising small, water filled pores and silt/clay sized solid particles below resolution (*Mixed*). For this purpose, the "trainable WEKA segmentation" plugin in ImageJ was used. It combines machine learning algorithms with selected image features to produce a classifier which is used for pixel-based segmentation. First, individual features were tested qualitatively on test slices to identify a feature set that was able to detect all three phases. Chosen features were "entropy" and "neighbours" using kernel sizes of σ=2 to 16. The plugin requires the user to manually draw traces of each phase on a 2D slice to train the classifier. The segmentation result is shown as an overlay on the original image allowing the user to add additional traces in an iterative process.

The performance was assessed semi-quantitatively in a representative test slice of 300 x 300 pixels. The major pores and selected primary mineral grains in the slice were manually segmented to produce ground truth images of the pore and solid phases, respectively. The performance of the WEKA process was tested by intersecting the segmentation result of each iteration with the ground truth image and measuring the fraction of correctly classified pixels (Figure S1 and Table S1). After 4 iterations the fraction of correctly identified pixels was 0.95 for the pore phase and 0.97 for the solid phase, respectively. Misclassified pixels were almost exclusively classified as mixed phase. We therefore note that this process might result in a small over-segmentation of the mixed phase. This was especially true for small structures. The minimum size of detectable pore space was $\gtrsim$ 5 µm.

For the full volume segmentation of each scan (Figure 2b), training regions were manually defined in 5 sub-slices of each 3D image, and used to generate a classifier. The resulting classifier was

applied slice by slice to the full image stack using an ImageJ macro. A 3D majority filter of 5x5x5 pixels was applied after segmentation to reduce noise. The final segmentation was compared with the ground-truth test slice and the performance was slightly worse, but the fraction of correctly identified pixels was still above 0.9, and most misclassified pixels were on edges between two phases.
